# Exploratory Analysis of Thromboinflammatory and Hematological Changes Following Electrolytic Femoral Vein Injury in Wistar Rats

**DOI:** 10.7759/cureus.116218

**Published:** 2026-09-14

**Authors:** Emma Eugenia Murariu-Gligor, Bogdan Cordoș, Andreea-Raluca Cozac-Szőke, Ovidiu S Cotoi, Carmen Căldăraru, Simona Mureșan

**Affiliations:** 1 Doctoral School of Medicine and Pharmacy, Institution Organizing University Doctoral Studies (IOSUD), George Emil Palade University of Medicine, Pharmacy, Science, and Technology of Târgu Mureș, Târgu Mureș, ROU; 2 Department of Internal Medicine IV, George Emil Palade University of Medicine, Pharmacy, Science, and Technology of Târgu Mureș, Târgu Mureș, ROU; 3 Internal Medicine Clinic, Mureș County Clinical Hospital, Târgu Mureș, ROU; 4 Center of Experimental and Imaging Studies, George Emil Palade University of Medicine, Pharmacy, Science, and Technology of Târgu Mureș, Târgu Mureș, ROU; 5 Department of Pathophysiology, George Emil Palade University of Medicine, Pharmacy, Science, and Technology of Târgu Mureș, Târgu Mureș, ROU; 6 Department of Pathology, Mureș County Clinical Hospital, Târgu Mureș, ROU

**Keywords:** animal model, anticoagulation, biomarker, electrolytic injury, granulocyte-to-lymphocyte ratio, myeloperoxidase, platelet-to-lymphocyte ratio, thromboinflammation, venous thrombosis

## Abstract

Introduction

Animal models provide controlled experimental settings for investigating thromboinflammatory processes and evaluating potential biomarkers. The aim of this study was to explore the hematological and inflammatory responses following an adapted electrolytic femoral vein injury protocol in laboratory rats. Particular emphasis was placed on identifying potential associations between blood parameters and histopathologically detected venous thrombosis, as well as comparison of two different anticoagulant regimens.

Methods

Twenty-four female Wistar rats (approximately 15 months old; age varied slightly in weeks between sequentially allocated groups) were allocated to a sham control group (n = 4), an untreated electrolytic injury control group (n = 4), and an electrolytic injury group treated with either enoxaparin or rivaroxaban (n = 8 each). Blood samples were collected at baseline (T0), one day (T1), and seven days (T7) post-intervention. Complete blood count (CBC) parameters, CBC-derived indices (granulocyte-to-lymphocyte ratio (GLR), platelet-to-lymphocyte ratio (PLR)), and plasma myeloperoxidase (MPO) levels were determined. Following euthanasia on day one (untreated group) or day seven (sham and treated groups), thrombus presence was assessed histopathologically.

Results

Seven days post-intervention, the leukocyte, granulocyte, and lymphocyte counts decreased, while the PLR increased significantly in all study groups. MPO increased significantly at T7 compared to baseline in the rivaroxaban group, while no significant changes were observed in the enoxaparin group. No significant between-treatment differences were observed in GLR, PLR, or MPO levels. Histopathological analysis confirmed thrombi occurrence in 6/20 animals (1/4 untreated and 5/16 treated). Exploratory ROC analysis suggested discriminatory potential of MPO, but not GLR or PLR, for histopathologically detected thrombus.

Conclusion

The adapted electrolytic femoral vein injury model was associated with dynamic changes in hematological and inflammatory parameters. MPO showed potential as a thromboinflammatory biomarker. However, given the limited detection of thrombus, these findings require validation in larger studies, using a more reliable thrombosis induction protocol.

## Introduction

The term "immunothrombosis" was first introduced by Engelmann and Massberg in 2013 [1] as “an independent process of innate immunity”, a physiological phenomenon occurring locally within the microvasculature, aimed at containing pathogens or harmful stimuli [1]. The concept of thromboinflammation had already been introduced earlier, primarily in reference to the involvement of platelets in inflammatory processes, such as in-stent restenosis after coronary artery angioplasty [2], or acute infections leading to increased likelihood of vascular thrombotic events [3]. However, in current usage, terminology has evolved to better reflect the complex, bidirectional interplay between coagulation and immunity. More specifically, inflammatory states may promote thrombus formation through immune-mediated mechanisms (immunothrombosis), while thrombus formation (whether or not driven by immunothrombosis) can further amplify inflammation (thromboinflammation) [4].

Leukocytes and platelets play a significant role in thrombosis both individually, as well as through their constant interactions. Platelets mediate venous thrombosis initiation by attaching to the vessel wall, where they interact with leukocytes and support further leukocyte recruitment to the site of thrombosis [5]. Leukocytes synthesize and release pro-inflammatory and procoagulant mediators, promoting thrombus formation; later, leukocytes get involved in thrombus resolution through phagocytosis and modulation of fibrinolysis [6]. Beyond initiating thrombus formation, research has shown that platelets can secrete certain coagulation factors (e.g., Von Willebrand factor, factors XIII, IX, VIII, V, fibrinogen, protein S), therefore also being actively involved in thrombus evolution, then resolution and venous wall remodeling [5].

This constant interplay between leukocytes and platelets may be indirectly reflected in the dynamics of complete blood count (CBC) cell lines and their derived ratios, such as neutrophil-to-platelet ratio (NLR), granulocyte-to-lymphocyte ratio (GLR), or platelet-to-lymphocyte ratio (PLR). While NLR has been extensively researched in the context of venous thromboembolism [7], GLR has been less reported, although it reflects the integrated granulocytic contribution to thrombosis, including eosinophil and basophil involvement. As granulocyte counts are routinely available in automated CBC systems and standardized across laboratories, the use of GLR has the benefit of easy reproducibility while being clinically translatable.

Myeloperoxidase (MPO) is a heme-containing enzyme, a member of the peroxidase-cyclooxygenase superfamily, found mostly in neutrophils and to a lower extent in monocytes [8,9], as MPO is usually lost when monocytes mature to macrophages [9]. MPO can also be of exosomal origin [10]. MPO is involved in NETosis, a process supporting immunothrombosis, innate to neutrophils, during which a fibrillar meshwork composed of decondensed chromatin associated with nuclear proteins (e.g., histones) and neutrophil-derived serine proteases (such as MPO) is released, forming the so-called “neutrophil extracellular traps” (NETs) [11,12]. MPO has both non-enzymatic effects (facilitating polymorphonuclear neutrophils’ recruitment) and enzymatic properties (contributing to innate immune defense mechanisms through generation of reactive oxygen species, with antimicrobial effects) [8]. However, although initially useful for innate immunity, dysregulation of MPO circulating levels leads to tissue damage [13]. Plasma MPO levels have been determined to be higher in venous thromboembolism patients compared to healthy subjects [14]. Efforts have been made to develop MPO inhibitors, both irreversible and reversible agents, although natural antioxidants such as polyphenols and flavonoids, e.g., quercetin, seem to already have a reversible inhibitory effect on MPO activity [15]. Previous research has also shown that heparin and low-molecular-weight heparin reduce the MPO binding to endothelial cells [16]. In an in vitro model of cancer-associated thrombosis based on prostate cancer cell lines, comparison of different anticoagulants showed that MPO partially inhibited low-molecular-weight heparins such as tinzaparin and dalteparin, but not the direct oral anticoagulant rivaroxaban [17].

Experimental animal models of venous thrombosis have significantly contributed to clarifying pathophysiological mechanisms underlying thrombosis, while also facilitating the development of therapeutic strategies [18]. The main murine models recommended involve either venous ligation, venous stenosis, or electrolytic induction of venous injury [19,20]. Although these models have provided important insights into thrombosis development, their performance may vary according to species, anatomical site, and experimental conditions.

An electrolytic injury protocol capable of inducing femoral vein thrombosis was proposed by Cooley et al. in mice [19,21], but to the best of our knowledge, further application to rat biology has not been investigated. Therefore, although this approach has been described in mice, its adaptation to rats and its reproducibility in this species remain to be established. This represents an important methodological gap, particularly for studies aiming to investigate hematological, inflammatory, and vascular responses in this model.

The present exploratory study aimed to adapt an electrolytic venous thrombosis model previously described in mice for use in Wistar rats. We investigated the hematological and inflammatory responses following electrolytic femoral vein injury, with particular focus on changes associated with the occurrence of histopathologically confirmed thrombi and comparisons of two different anticoagulant treatments.

## Materials and methods

Study design

A prospective, interventional, exploratory animal study was conducted in 2023 in accordance with the ARRIVE guidelines, adhering to the principles of Replacement, Reduction, and Refinement (3Rs). Ethical approval was obtained from the relevant institutional and national regulatory bodies, namely the Scientific Research Ethics Committee of George Emil Palade University of Medicine, Pharmacy, Science, and Technology of Târgu Mureş (Decision nr. 1881 from October 12, 2022), and the Veterinary and Food Safety Authority Mureş - Animal Health and Welfare Official Control Service (Project Authorization nr. 58 from March 6, 2023).

The primary objective of this exploratory study was to characterize hematological and inflammatory responses following an adapted electrolytic femoral vein injury procedure in laboratory rats. The outcomes assessed included CBC-derived inflammatory indices (GLR and PLR), plasma MPO levels, and histopathological detection of intraluminal thrombotic material. Secondary exploratory analyses examined whether hematological and inflammatory responses varied according to thrombus status and according to the anticoagulant treatment administered.

Experimental Animals

The study included 24 female Wistar rats (Rattus norvegicus), aged approximately 15 months, obtained from the university's animal facility, where they were bred and raised. Animals were group-housed (to support their social behavior) in standard polycarbonate cages under controlled conditions (12 h light/dark cycle), with a standardized diet and water provided ad libitum. All procedures and animal handling were carried out by trained personnel, in compliance with animal welfare regulations. Humane endpoints, predefined in accordance with institutional animal care guidelines (including severe weight loss, persistent signs of pain or distress, impaired mobility, or severe postoperative complications requiring euthanasia), were not met throughout the study.

Baseline health status was assessed clinically and supported by CBC analysis from caudal vein sampling. For screening purposes, CBC values were compared with the reference ranges available in the analyzer's rat mode at the time of analysis. All 24 animals met the available screening criteria and were therefore considered eligible for the study procedures.

The sample size was determined based on previous experience with similar animal models and practical considerations, including ethical principles to minimize animal use. Animals were allocated into three groups: (1) sham control (n = 4), (2) electrolytic injury model control (untreated, n = 4), and (3) electrolytic injury treated group (n = 16) subdivided into two anticoagulation arms (enoxaparin and rivaroxaban, n = 8 each). Control groups were used to ensure procedural consistency, while the treatment group was used to assess potential treatment-related effects. Randomization was not implemented because the study involved the initial implementation of a microsurgical injury procedure at our center. Animals were therefore allocated sequentially by study group, starting with sham control, followed by the electrolytic injury control group, and subsequently proceeding to the enoxaparin- and rivaroxaban-treated groups. This approach was chosen to account for the operator learning curve and to promote consistency in the application of the experimental procedures within each group. Animals were group-housed by study group, with individual identification ensured by ear-notching. Baseline body weight was recorded for animals in the anticoagulant-treated groups for dose calculation; mean baseline weight was 294 ± 28 g in the enoxaparin-treated group and 296 ± 32 g in the rivaroxaban-treated group. Corresponding baseline weight data were not recorded for the control groups. Blinding was applied for outcome assessment when feasible (e.g., histopathological analysis), while procedural steps could not be blinded.

**Figure 1:**
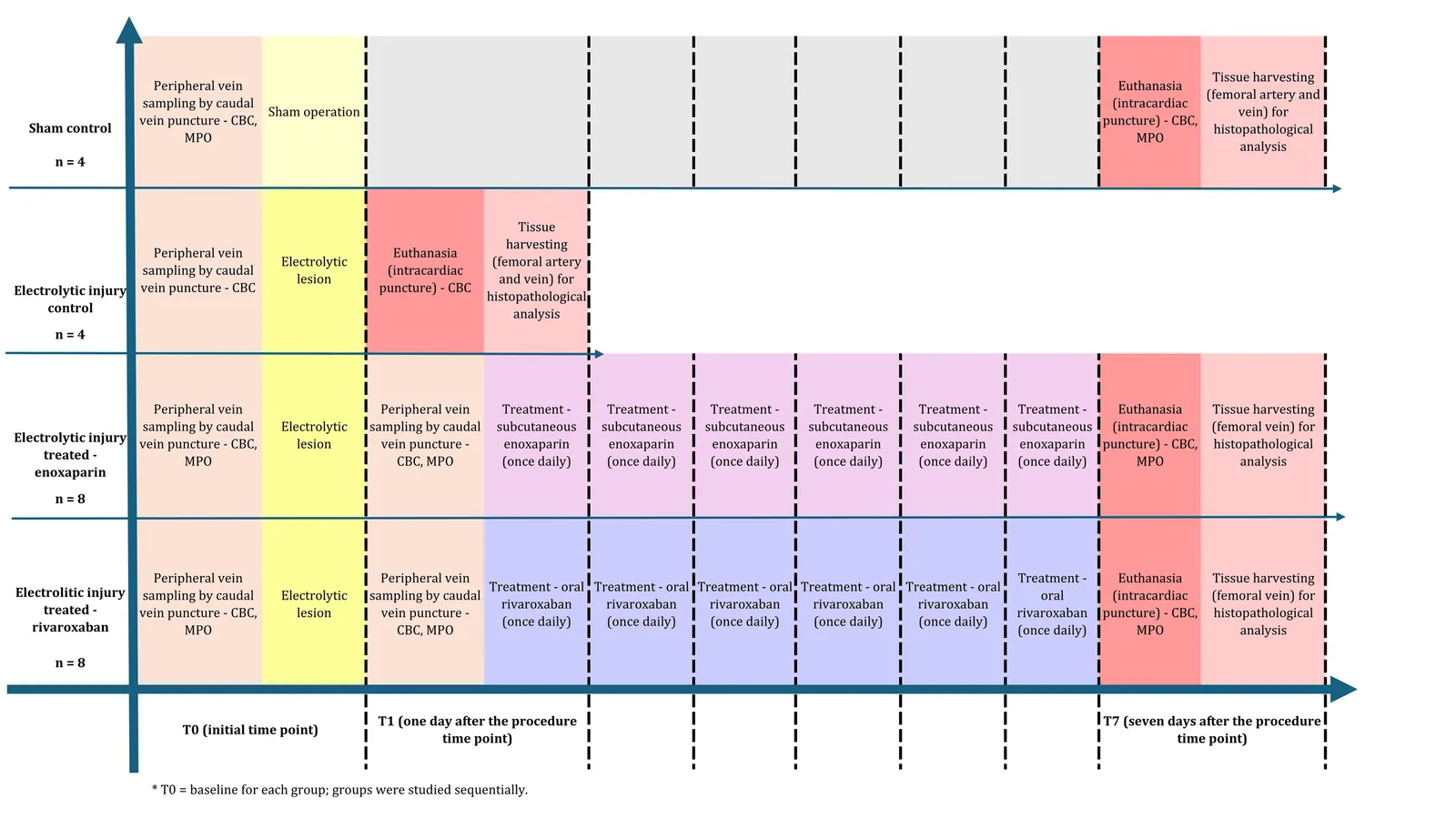
Schematic timeline of the experimental procedures Blood sampling, intervention/treatment, euthanasia, and tissue harvesting are shown relative to each group's baseline assessment (T0). Because the experimental groups were implemented sequentially, the timelines represent relative study time within each group and do not indicate simultaneous calendar time. CBC: complete blood count; MPO: myeloperoxidase. Figure created using Microsoft Excel for Microsoft 365 (Version 2608).

A general overview of the study design is provided in Figure 1.

All procedures (blood sampling, surgical procedures including application of electrolytic injury, injectable treatments, euthanasia) were conducted under isoflurane anesthesia (Anesteran, Rompharm Company S.R.L., Romania). Post-procedural analgesia consisted of subcutaneous tramadol (0.1 mL, 50 mg/mL; Tramadol, KRKA, D.D., Novo Mesto, Slovenia) to minimize animal pain and distress.

Electrolytic Injury Model and Experimental Groups

**Figure 2:**
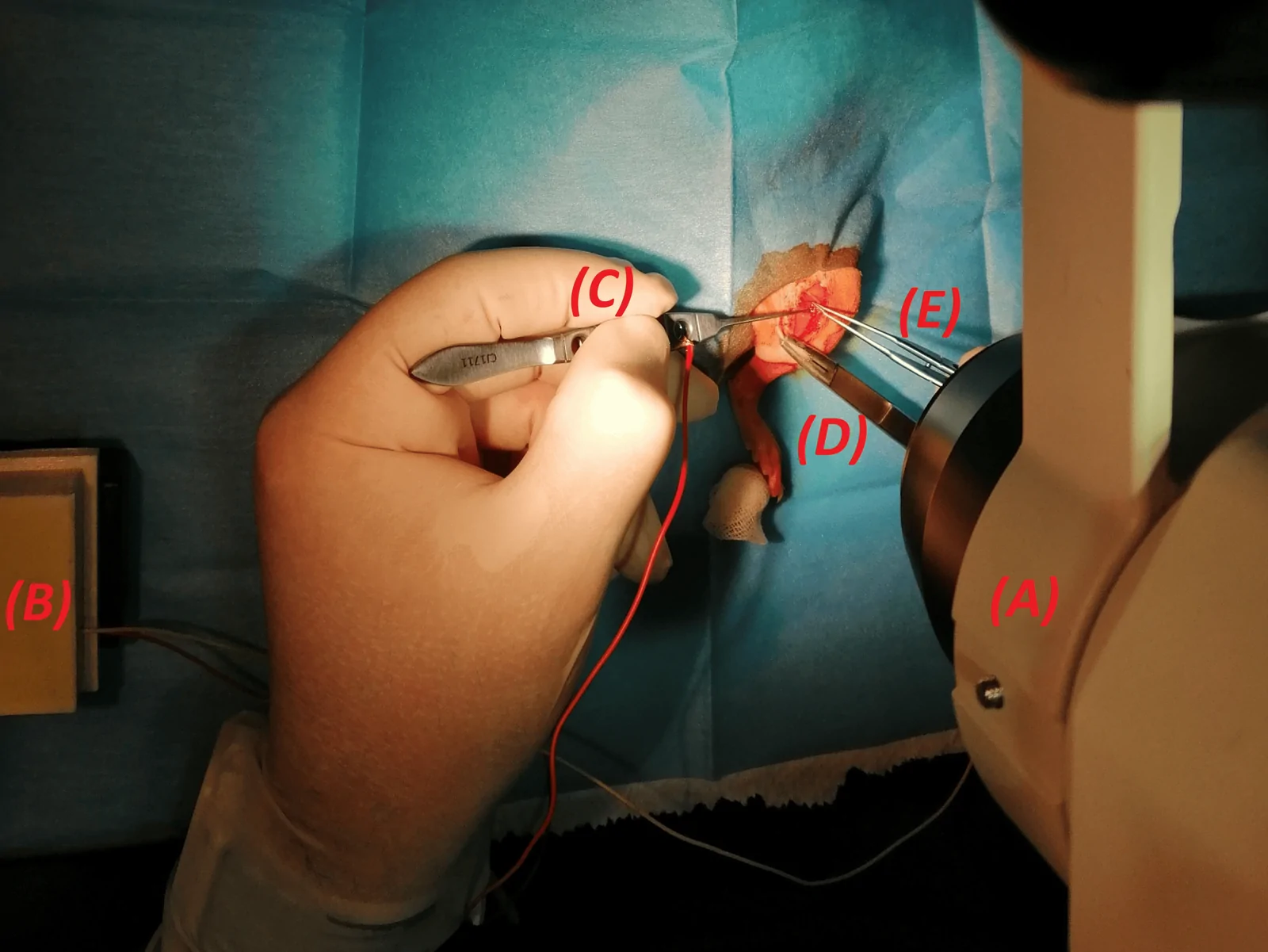
Venous electrolytic injury model setup Following thorough skin antisepsis of the lower left limb with povidone-iodine (Betadine) and isolation of the limb within a sterile surgical field, a skin incision was performed, and the femoral neurovascular bundle was carefully dissected. Under anesthesia and microscopic visualization (A), a direct current of 3 volts was applied to the exposed surface of the left femoral vein for a duration of 90 seconds. (B) The source of continuous electrical current. (C) The forceps connected to the positive terminal of the electrical circuit (red wire) was used to clamp a 10/0 needle, and the blunt end of the needle was gently applied to the exposed surface of the vein. (D) The forceps connected to the negative pole of the electrical circuit (yellow wire) was secured by clamping onto the tegument. (E) A third forceps, equipped with a suture wire, was employed to gently secure and isolate the adjacent neurovascular structures (nerve and artery) from direct exposure to the electrical current during the procedure.

Surgical exposure of the femoral neurovascular bundle was performed via an inguinal approach. In the sham group, no electrolytic injury was induced. In the model control and treatment groups, an electrolytic protocol was applied, adapted for Wistar rat use from the recommendations outlined in the 2019 consensus on mouse models of venous thrombosis [19], by increasing the applied voltage (from 1.5 V to 3 V) and extending the duration of current delivery to 90 seconds (Figure 2).

Anticoagulant Treatment

Post-intervention protocols differed by group. The electrolytic injury model control group received no anticoagulation and was euthanized one day after the procedure. The treatment group (n = 16) was subdivided into two anticoagulation arms: enoxaparin (n = 8) and rivaroxaban (n = 8). Anticoagulation began one day after the electrolytic injury and was continued for a total of six days. The enoxaparin-treated arm received subcutaneous enoxaparin (Clexane, Sanofi Romania SRL) at 10.5 mg/kg/day. In the rivaroxaban arm, voluntary ingestion was encouraged by incorporating the drug into a palatable mixture: the medication was combined with Nutella (a highly preferred food for laboratory rats) and excipients such as starch and lactose to increase the volume and consistency. Oral rivaroxaban (Xarelto, Bayer AG) was administered via Nutella-based pellets (0.75 mg rivaroxaban per 281 mg pellet). Voluntary oral ingestion was preferred to oral gavage to minimize handling stress and to prevent possible mucosal injury associated with gavage in anticoagulated animals. Nutella preconditioning was started two days prior and continued daily; throughout every session of oral medication administration, each animal was temporarily moved to an individual cage, where pellet ingestion took place in a supervised manner, followed by return to the group cage with a reward. Sham and treatment groups were euthanized on day seven.

Blood Sampling and Laboratory Analyses

Blood samples were collected at predefined time points: baseline, before surgery (T0), day one post-intervention (T1), and day seven post-intervention (T7). Caudal vein sampling under anesthesia was limited to 0.5 mL, while cardiac puncture at euthanasia allowed collection of 1 mL. For peripheral sampling, tails were pre-warmed (~40 °C) to induce vasodilation. For both peripheral and cardiac puncture, heparinized needles and 0.5 mL or 1 mL K₂-EDTA collecting tubes were used to prevent clotting. The treatment group sampling at T1 was performed prior to the first anticoagulant dose. Sampling occurred as follows: T0 - all groups via caudal vein; T1 - sham and treatment groups via caudal vein and electrolytic injury model control via cardiac puncture; T7 - sham and treatment groups via cardiac puncture.

All blood samples underwent analysis for CBC determination (Mindray BC-5000 Vet hematology analyzer, Mindray Bio-Medical Electronics Co., Ltd., China); CBC-derived indices, GLR, and PLR were subsequently calculated. Remaining blood was centrifuged (1000×g, 15 minutes) to obtain plasma, which was stored at −80°C and later analyzed for MPO levels using the Abbexa Low Sample Volume Rat MPO ELISA kit (Abbexa Ltd, Cambridge, UK; catalog no. abx055219, lot no. E2405285K). Before analysis, plasma was thawed at room temperature, diluted 1:10 in PBS (0.01 mol/L, pH 7.0-7.2), and assayed in triplicate following the manufacturer’s protocol. Optical densities were measured at 450 nm, and concentrations were calculated from a sigmoid calibration curve, corrected for the dilution factor. Assay sensitivity was <0.13 ng/mL, with a reference range of 0.312-20 ng/ml. Final MPO concentrations were calculated by multiplying measured values by the dilution factor of 10.

Euthanasia was performed at designated endpoints via intracardiac puncture under anesthesia, with terminal blood collection.

Histopathological Assessment

Femoral vein tissue samples were collected and preserved in 10% formalin, followed by paraffin embedding. Sections (3 μm) obtained from the paraffin blocks were mounted on glass slides and routinely stained with hematoxylin and eosin (H&E). Histopathological assessment was independently performed by two pathologists who were blinded to the experimental group. Thrombus detection was based on the identification of an intraluminal or wall-adherent thrombotic mass rich in fibrin, erythrocytes, and inflammatory cells, consistent with venous thrombosis. Any discrepancies between the two assessments were resolved by consensus. For the purposes of the present study, which focuses on blood biomarkers and their associations with histopathologically detected thrombus, animals were classified according to whether thrombus was detected histopathologically at the time of euthanasia. Detailed characterization and interpretation of the histopathological findings are addressed separately in a published companion manuscript [22].

Statistical analysis

Data were organized using Microsoft Excel for Microsoft 365 (Version 2608). Statistical analyses were performed using MedCalc® Statistical Software version 23.5.0 (MedCalc Software Ltd, Ostend, Belgium; https://www.medcalc.org; 2026).

Normality of data distribution was assessed using the Shapiro-Wilk test. Variables that met the assumption of normality were reported as mean ± standard deviation, while non-normally distributed variables were presented as median with interquartile range (IQR).

Within-group comparisons over time were performed using repeated-measures analysis of variance (ANOVA) or the Friedman test, respectively, for data available at three time points (T0, T1, T7). For variables assessed at two time points, within-group comparisons were performed using paired t-tests for normally distributed data or Wilcoxon signed-rank tests for non-normally distributed data. Normally distributed repeated measures at three time points were analyzed using repeated-measures ANOVA, followed by post-hoc pairwise comparisons using paired t-tests. Non-normally distributed repeated measures at three time points were analyzed using the Friedman test, followed by post-hoc pairwise comparisons using Wilcoxon signed-rank tests. If the repeated-measures ANOVA or Friedman test reached statistical significance but post-hoc pairwise comparisons were not significant after adjustment, the result was interpreted as indicating an overall difference among the repeated measures without sufficient evidence to identify specific pairwise differences.

Between-group comparisons at each time point were performed using one-way ANOVA for three or more groups of normally distributed data, or the independent-samples t-test for two groups of normally distributed data. Non-normally distributed data from two independent groups were analyzed using the Mann-Whitney U test. Where appropriate, one-way ANOVA was followed by post-hoc pairwise comparisons using the Scheffé test.

Receiver operating characteristic (ROC) curve analyses were performed using biomarker values at T7 to explore their discriminatory ability regarding histopathologically detected thrombus.

Statistical significance was set at p < 0.05 for overall analyses. For post-hoc pairwise comparisons, unadjusted p-values were presented, with Bonferroni correction applied using an adjusted significance threshold of p < 0.017 for three comparisons.

## Results

Hematological and inflammatory changes following the procedure

For each experimental group, mean or median values of CBC parameters at each assigned time point are presented in Table 1. Comparisons between time points are presented in Table 2.

**Table 1:** Hematological parameters in the experimental groups Normality of data distribution was assessed using the Shapiro–Wilk test. Data are presented as mean ± standard deviation or median (interquartile range (IQR)), as appropriate. CBC: complete blood count; WBC #: white blood cell count; Gran #: granulocyte count; Lym #: lymphocyte count; Mon #: monocyte count; RBC: red blood cell count; Hgb: hemoglobin; Htc: hematocrit; PLT: platelet count; ul: microliter; GLR: granulocyte-to-lymphocyte ratio; PLR: platelet-to-lymphocyte ratio; T0: initial time point; T1: one day after the intervention time point; T7: seven days after the intervention time point; NA: not applicable; vs: versus.

CBC parameters/time point	Sham control (n = 4)		Electrolytic injury control group (n = 4)		Electrolytic injury treated group - enoxaparin (n = 8)			Electrolytic injury treated group - rivaroxaban (n = 8)		
	T0	T7	T0	T1	T0	T1	T7	T0	T1	T7
WBC # (*10^3 /ul)	6.34 ± 1.02	2.19 ± 0.41	8.72 ± 2.67	3.17 ± 0.73	6.99 ± 2.67	5.69 (5.25-6.47)	2.41 ± 0.76	5.81 ± 1	6.26 ± 0.54	2.75 ± 0.94
Gran # (*10^3 /ul)	2.33 ± 0.6	0.67 ± 0.11	3.02 ± 1.23	1.37 ± 0.45	2.24 ± 1.06	2.56 (1.9-3.03)	0.87 (0.71-1.16)	2.09 ± 0.71	2.45 ± 0.4	0.97 ± 0.54
Lym # (*10^3 /ul)	3.35 ± 0.39	1.4 ± 0.34	5.22 ± 1.73	1.62 ± 0.33	4.13 ± 1.54	3.26 (2.62-3.4)	1.27 ± 0.51	3.19 ± 0.68	3.42 ± 0.7	1.57 ± 0.4
Mon # (*10^3 /ul)	0.66 ± 0.1	0.1 (0.09-0.16)	0.48 ± 0.14	0.18 ± 0.06	0.5 (0.43-0.82)	0.36 (0.29-0.72)	0.21 ± 0.07	0.52 ± 0.27	0.39 ± 0.08	0.21 ± 0.1
RBC (*10^6 /ul)	6.46 ± 0.22	5.98 ± 0.42	7.51 ± 0.66	6.97 ± 0.42	6.97 ± 0.58	7.28 ± 0.28	7.23 (6.97-7.43)	6.88 ± 0.29	6.7 ± 0.4	6.85 ± 0.48
Hgb (g/dL)	13.68 ± 0.43	12.75 ± 0.69	13.1 ± 0.65	12.13 ± 0.52	12.24 ± 0.53	12.44 ± 0.67	12.1 (11.65-12.3)	12.1 ± 0.49	11.7 ± 0.4	11.61 ± 0.6
Htc (%)	34.38 ± 1.63	32.78 ± 2.79	38.4 ± 2.54	35.93 ± 1.68	12.24 ± 0.53	36.61 ± 1.85	36.55 (35.2-37.5)	34.95 ± 1.6	34.2 ± 1.68	34.66 ± 1.62
PLT (*10^3 /ul)	696 ± 313.48	1026.25 ± 133.71	1099.75 ± 58.01	1164.5 ± 67.69	1055.75 ± 177.86	1138 (1093.5-1189.5)	1368.38 ± 160.15	979.38 ± 152	942.5 ± 142.51	1301 (1165-1437)
GLR	0.69 ± 0.13	0.49 ± 0.11	0.61 ± 0.26	0.85 ± 0.21	0.55 ± 0.15	0.71 (0.53-1)	0.79 ± 0.23	0.69 ± 0.31	0.76 ± 0.25	0.61 ± 0.27
PLR	204.9 ± 83.68	762.38 ± 175.83	228.27 ± 72.01	744.01 ± 171.96	273.69 ± 67.95	364.89 (335.19-411.92)	1232.41 ± 512.16	322.11 ± 99.44	283.42 ± 63.03	787.85 ± 445.18

**Table 2:** Intra-group comparative analysis of hematological parameters across assessed time points within each experimental group Normality of data distribution was assessed using the Shapiro–Wilk test. Normally distributed repeated measures were analyzed using repeated-measures ANOVA, followed by post-hoc pairwise comparisons using paired t-tests. Non-normally distributed repeated measures were analyzed using the Friedman test, followed by post-hoc pairwise comparisons using Wilcoxon signed-rank tests. If the repeated-measures ANOVA or Friedman test reached statistical significance but post-hoc pairwise comparisons were not significant after adjustment, the result was interpreted as indicating an overall difference among the repeated measures without sufficient evidence to identify specific pairwise differences. Statistical significance was set at p < 0.05 for overall analyses. For post-hoc pairwise comparisons, unadjusted p-values are presented, with Bonferroni correction applied using an adjusted significance threshold of p < 0.017 for three comparisons. CBC: complete blood count; WBC #: white blood cell count; Gran #: granulocyte count; Lym #: lymphocyte count; Mon #: monocyte count; RBC: red blood cell count; Hgb: hemoglobin; Htc: hematocrit; PLT: platelet count; ul: microliter; GLR: granulocyte-to-lymphocyte ratio; PLR: platelet-to-lymphocyte ratio; T0: initial time point; T1: one day after the intervention time point; T7: seven days after the intervention time point; NA: not applicable; ns: non-significant; vs: versus.

Group	CBC parameters	Time point comparison	Statistical test	Test statistic	p-value	Post-hoc comparison (Bonferroni-adjusted significance threshold: p < 0.017)
Sham control	WBC # (*10^3 /ul)	T0 vs T7	Paired t-test	t(3) = -9.055	p = 0.003	NA
	Gran # (*10^3 /ul)	T0 vs T7	Paired t-test	t(3) = -5.992	p = 0.009	NA
	Lym # (*10^3 /ul)	T0 vs T7	Paired t-test	t(3) = -10.122	p = 0.002	NA
	Mon # (*10^3 /ul)	T0 vs T7	Paired Wilcoxon signed-rank test	-	ns (p = 0.125)	NA
	RBC (*10^6 /ul)	T0 vs T7	Paired t-test	t(3) = -1.703	ns (p = 0.187)	NA
	Hgb (g/dL)	T0 vs T7	Paired t-test	t(3) = -2.496	ns (p = 0.088)	NA
	Htc (%)	T0 vs T7	Paired t-test	t(3) = -0.971	ns (p = 0.403)	NA
	PLT (*10^3 /ul)	T0 vs T7	Paired t-test	t(3) = 3.514	p = 0.039	NA
	GLR	T0 vs T7	Paired t-test	t(3) = -3.563	p = 0.038	NA
	PLR	T0 vs T7	Paired t-test	t(3) = 6.832	p = 0.006	NA
Electrolytic injury control	WBC # (*10^3 /ul)	T0 vs T1	Paired t-test	t(3) = -3.48	p = 0.04	NA
	Gran # (*10^3 /ul)	T0 vs T1	Paired t-test	t(3) = -2.102	ns (p = 0.126)	NA
	Lym #(*10^3 /ul)	T0 vs T1	Paired t-test	t(3) = -3.744	p = 0.033	NA
	Mon # (*10^3 /ul)	T0 vs T1	Paired t-test	t(3) = -3.245	p = 0.048	NA
	RBC (*10^6 /ul)	T0 vs T1	Paired t-test	t(3) = -1.447	ns (p = 0.244)	NA
	Hgb (g/dL)	T0 vs T1	Paired t-test	t(3) = -1.687	ns (p = 0.190)	NA
	Htc (%)	T0 vs T1	Paired t-test	t(3) = -1.304	ns (p = 0.283)	NA
	PLT (*10^3 /ul)	T0 vs T1	Paired t-test	t(3) = 1.615	ns (p = 0.205)	NA
	GLR	T0 vs T1	Paired t-test	t(3) = 1.243	ns (p = 0.302)	NA
	PLR	T0 vs T1	Paired t-test	t(3) = 4.635	p = 0.019	NA
Electrolytic injury – enoxaparin treated	WBC # (*10^3 /ul)	T0 vs T1 vs T7	Friedman test	χ²(2) = 22.87	p < 0.001	Wilcoxon signed rank test: T0 vs T1: ns (p = 0.674); T0 vs T7: p = 0.008; T1 vs T7: p = 0.008
	Gran # (*10^3 /ul)	T0 vs T1 vs T7	Friedman test	χ²(2) = 22.87	p < 0.001	Wilcoxon signed rank test: T0 vs T1: ns (p = 0.461); T0 vs T7: p = 0.008; T1 vs T7: p = 0.008
	Lym #(*10^3 /ul)	T0 vs T1 vs T7	Friedman test	χ²(2) = 14.33	p < 0.001	Wilcoxon signed rank test: T0 vs T1: ns (p = 0.078); T0 vs T7: p = 0.008; T1 vs T7: p = 0.016
	Mon # (*10^3 /ul)	T0 vs T1 vs T7	Friedman test	χ²(2) = 22.87	p < 0.001	Wilcoxon signed rank test: T0 vs T1: ns (p = 0.293); T0 vs T7: p = 0.008; T1 vs T7: p = 0.012
	RBC (*10^6 /ul)	T0 vs T1 vs T7	Friedman test	χ²(2) = 2.38	p = 0.13	NA
	Hgb (g/dL)	T0 vs T1 vs T7	Friedman test	χ²(2) = 1.78	p = 0.2	NA
	Htc (%)	T0 vs T1 vs T7	Friedman test	χ²(2) = 0.86	p = 0.44	NA
	PLT (*10^3 /ul)	T0 vs T1 vs T7	Friedman test	χ²(2) = 4.49	p = 0.031	Wilcoxon signed rank test: T0 vs T1: ns (p = 0.109); T0 vs T7: p = 0.016; T1 vs T7: ns (p = 0.039)
	GLR	T0 vs T1 vs T7	Friedman test	χ²(2) = 0.86	p = 0.444	NA
	PLR	T0 vs T1 vs T7	Friedman test	χ²(2) = 22.87	p < 0.001	Wilcoxon signed rank test: T0 vs T1: p = 0.016; T0 vs T7: p = 0.008; T1 vs T7: p = 0.016
Electrolytic injury – rivaroxaban treated	WBC # (*10^3 /ul)	T0 vs T1 vs T7	Repeated measures ANOVA	F(2,14) = 41.75	p < 0.001	Paired t-test: T0 vs T1: ns (p = 0.344); T0 vs T7: p < 0.001; T1 vs T7: p < 0.001
	Gran # (*10^3 /ul)	T0 vs T1 vs T7	Repeated measures ANOVA	F(2,14) = 40.9	p < 0.001	Paired t-test: T0 vs T1: ns (p = 0.077); T0 vs T7: p < 0.001; T1 vs T7: p < 0.001
	Lym # (*10^3 /ul)	T0 vs T1 vs T7	Repeated measures ANOVA	F(2,14) = 31.53	p < 0.001	Paired t-test: T0 vs T1: ns (p = 0.400); T0 vs T7: p < 0.001; T1 vs T7: p < 0.001
	Mon # (*10^3 /ul)	T0 vs T1 vs T7	Repeated measures ANOVA	F(1.52, 10.63) = 6.12	p = 0.022	Paired t-test: T0 vs T1: ns (p = 0.227); T0 vs T7: ns (p = 0.020); T1 vs T7: ns (p = 0.019);
	RBC (*10^6 /ul)	T0 vs T1 vs T7	Repeated measures ANOVA	F(2,14) = 0.54	p = 0.593	NA
	Hgb (g/dL)	T0 vs T1 vs T7	Repeated measures ANOVA	F(2, 14) = 1.99	p = 0.173	NA
	Htc (%)	T0 vs T1 vs T7	Repeated measures ANOVA	F(2,14) = 0.36	p = 0.706	NA
	PLT (*10^3 /ul)	T0 vs T1 vs T7	Friedman test	χ²(2) = 5.11	p = 0.022	Wilcoxon signed rank test: T0 vs T1: ns (p = 0.726); T0 vs T7: ns (p = 0.195); T1 vs T7: ns (p = 0.195)
	GLR	T0 vs T1 vs T7	Repeated measures ANOVA	F(2,14) = 2.44	p = 0.123	NA
	PLR	T0 vs T1 vs T7	Repeated measures ANOVA	F(1.09, 7.60) = 9.71	p = 0.014	Paired t-test: T0 vs T1: ns (p = 0.341); T0 vs T7: ns (p = 0.022); T1 vs T7: p = 0.012

In the sham control group, leukocyte, granulocyte, and lymphocyte counts, as well as GLR, decreased significantly, while platelet count and PLR increased significantly at T7 compared with T0.

In the electrolytic injury control group, leukocyte, lymphocyte, and monocyte counts decreased significantly, while PLR increased significantly at T1 compared with T0. No significant variation was observed in platelet count or GLR between T0 and T1.

In the enoxaparin group, leukocyte, granulocyte, lymphocyte, and monocyte counts decreased significantly at T7 compared with both T0 and T1, with no significant differences between T0 and T1. Platelet count increased significantly at T7 compared with T0. GLR did not vary significantly across the three time points, whereas PLR increased significantly from T0 to T1 and T7.

In the rivaroxaban group, leukocyte, granulocyte, and lymphocyte counts decreased significantly at T7 compared with both T0 and T1, with no significant differences between T0 and T1. Platelet count and GLR did not show significant changes over time. PLR increased significantly at T7 compared with T1, with no significant difference between T0 and T1 or between T0 and T7.

For each experimental group, mean MPO levels at every assigned time point and the corresponding statistical comparisons between time points are presented in Table 3.

**Table 3:** Intra-group comparative analysis of myeloperoxidase levels across assessed time points within each experimental group Normality of data distribution was assessed using the Shapiro–Wilk test. Data are presented as mean ± standard deviation. Normally distributed repeated measures were analyzed using repeated-measures ANOVA, followed by post-hoc pairwise comparisons using paired t-tests. If the repeated-measures ANOVA reached statistical significance but post-hoc pairwise comparisons were not significant after adjustment, the result was interpreted as indicating an overall difference among the repeated measures without sufficient evidence to identify specific pairwise differences. Statistical significance was set at p < 0.05 for overall analyses. For post-hoc pairwise comparisons, unadjusted p-values are presented, with Bonferroni correction applied using an adjusted significance threshold of p < 0.017 for three comparisons. MPO: myeloperoxidase; T0: initial time point; T1: one day after the intervention time point; T7: seven days after the intervention time point; NA: not applicable; ns: non-significant; vs: versus.

Group	MPO (ng/mL)	Time point	Time point comparison	Statistical test	Test statistic	p-value	Post-hoc comparison Bonferroni-adjusted significance threshold: p < 0.017
Sham control (n = 4)	85.5 ± 13.52	T0	T0 vs T7	Paired t-test	t(3) = 2.76	ns (p = 0.07)	NA
	100.04 ± 15.3	T7					
Electrolytic injury - enoxaparin treated (n = 8)	68.48 ± 14.38	T0	T0 vs T1 vs T7	Repeated measures ANOVA	F(1.49, 10.44) = 6.05	p = 0.024	Paired t-test: T0 vs T1: ns (p = 0.072); T0 vs T7: ns (p = 0.028); T1 vs T7: ns (p = 0.084)
	76.71 ± 6.67	T1					
	85.80±12.52	T7					
Electrolytic injury - rivaroxaban treated (n = 8)	57.74±15.52	T0	T0 vs T1 vs T7	Repeated measures ANOVA	F(1.88, 13.13) = 11.42	p = 0.002	Paired t-test: T0 vs T1: p = 0.006; T0 vs T7: p = 0.002; T1 vs T7: ns (p = 0.492)
	76.36±12.13	T1					
	80.48 ± 12.25	T7					

MPO levels did not vary significantly between T0 and T7 in the sham control group. In the enoxaparin treated group, repeated-measures ANOVA demonstrated a significant time effect (F(1.49, 10.44) = 6.05, p = 0.024); however, post-hoc paired t-tests did not identify significant differences between any pair of time points (p > 0.017). In the rivaroxaban treated group, repeated-measures ANOVA showed a significant time effect (F(1.88, 13.13) = 11.42, p = 0.002), with post-hoc paired t-tests showing significantly higher MPO levels at T7 compared with T0 (p = 0.002) and at T1 compared with T0 (p = 0.006).

Exploratory analysis of hematological and inflammatory changes according to anticoagulant treatment and histopathologically detected thrombus

Comparison of GLR, PLR and MPO levels between experimental groups at the same time points is presented in Table 4.

**Table 4:** Between-groups comparative analysis of GLR, PLR, and MPO at the assessed time points The number of groups included in each comparison reflects the availability of measurements at the respective time point and the use of comparable sampling procedures. Comparisons were performed among two, three, or four groups, as applicable. The sham control and electrolytic injury control groups were excluded from comparisons at time points for which measurements were not available; additionally, the electrolytic injury control group was excluded from T1 comparisons because a different sampling procedure was used. Normality of data distribution was assessed using the Shapiro–Wilk test. Normally distributed independent samples were analyzed using the independent-samples t-test (for two-group comparisons) or one-way ANOVA (for three or more groups), followed by post-hoc pairwise comparisons using the Scheffé test where appropriate. Non-normally distributed independent samples were analyzed using the Mann–Whitney U test. Statistical significance was set at p < 0.05 for overall analyses. GLR: granulocyte-to-lymphocyte ratio; PLR: platelet-to-lymphocyte ratio; MPO: myeloperoxidase; T0: initial time point; T1: one day after the intervention time point; T7: seven days after the intervention time point; NA: not applicable; ns: non-significant; vs: versus.

Parameter	Time point	Groups compared	Statistical test	Test statistic	p-value	Post-hoc comparison
GLR	T0	sham vs injury control vs enoxaparin treated vs rivaroxaban treated	One-way ANOVA	F(3,20) = 0.640	ns (p = 0.598)	NA
	T1	enoxaparin treated vs rivaroxaban treated	Mann-Whitney test (independent samples)	Mann-Whitney U = 32	ns (p = 1.00)	NA
	T7	sham vs enoxaparin treated vs rivaroxaban treated	One-way ANOVA	F(2,17) = 2.511	ns (p = 0.111)	NA
PLR	T0	sham vs injury control vs enoxaparin treated vs rivaroxaban treated	One-way ANOVA	F(3,20) = 2.208	ns (p=0.119)	NA
	T1	enoxaparin treated vs rivaroxaban treated	Mann-Whitney test (independent samples)	Mann-Whitney U = 14	ns (p = 0.065)	NA
	T7	sham vs enoxaparin treated vs rivaroxaban treated	One-way ANOVA	F(2,17) = 2.530	ns (p = 0.109)	NA
MPO (ng/mL)	T0	sham vs enoxaparin treated vs. rivaroxaban treated	One-way ANOVA	F(2,17) = 4.766	p = 0.023	Scheffé test: rivaroxaban treated vs sham control: p < 0.05; other pairwise comparisons: ns
	T1	enoxaparin treated vs rivaroxaban treated	Independent samples t-test	t(14) = −0.073	ns (p = 0.943)	NA
	T7	sham vs enoxaparin treated vs rivaroxaban treated	One-way ANOVA	F(2,17) = 3.062	ns (p = 0.073)	NA

For GLR and PLR, comparisons at T1 with the untreated injury control were not performed because blood samples were obtained using different sampling procedures. GLR and PLR did not differ significantly among the available experimental groups at any assessed time points.

Comparison of MPO levels among the available groups at baseline (T0) showed a significant overall difference among groups (one-way ANOVA, p = 0.023). Post-hoc Scheffé analysis identified significantly lower MPO levels in the rivaroxaban treated group compared with the sham control group, whereas the other pairwise comparisons were not significant. MPO measurements were not available for the electrolytic injury control group because of limited assay resources. Comparisons at T1 and T7 did not identify any significant differences among the available groups.

Histopathological analysis (H&E staining) detected intraluminal thrombotic material in 6 out of 20 samples obtained from animals that underwent the electrolytic procedure, including one of four animals in the untreated electrolytic injury control group at T1, and five of 16 animals in the treated groups at T7. Based on this assessment, animals within the electrolytic injury treated groups with histopathologically detected thrombus at T7 were compared to those without detectable thrombus, regardless of anticoagulant treatment allocation. No significant differences in GLR, PLR, or MPO levels were observed between subjects with and without histologically detected thrombus seven days post-electrolytic injury (Table 5).

**Table 5:** Comparison of mean GLR, PLR and MPO levels in animals with histopathologically detected thrombus versus no detectable thrombus at T7 Data are presented as mean ± standard deviation. Normally distributed data were analyzed using an independent samples t-test. Statistical significance was set at p < 0.05. GLR: granulocyte-to-lymphocyte ratio; PLR: platelet-to-lymphocyte ratio; MPO: myeloperoxidase; ns: non-significant; T7: seven days after the intervention time point.

	Thrombus not detected on histopathological analysis at T7 (n = 11)	Thrombus detected on histopathological analysis at T7 (n = 5)	Statistical test	Test statistic	p-value
GLR	0.64 ± 0.26	0.84 ± 0.23	Independent samples t test	t(14) = 1.493	ns (p = 0.16)
PLR	917.86 ± 526.84	1213.13 ± 484.43	Independent samples t test	t(14) = -1.063	ns (p = 0.31)
MPO	86.55 ± 12.86	75.66 ± 7.05	Independent samples t test	t(14) = 1.755	ns (p = 0.1)

Exploratory receiver operating characteristic (ROC) curve analysis was then performed to assess the discriminatory ability of GLR, PLR and plasma MPO measured at T7 for histopathologically detected thrombus and to identify candidate cutoff values.

**Figure 3:**
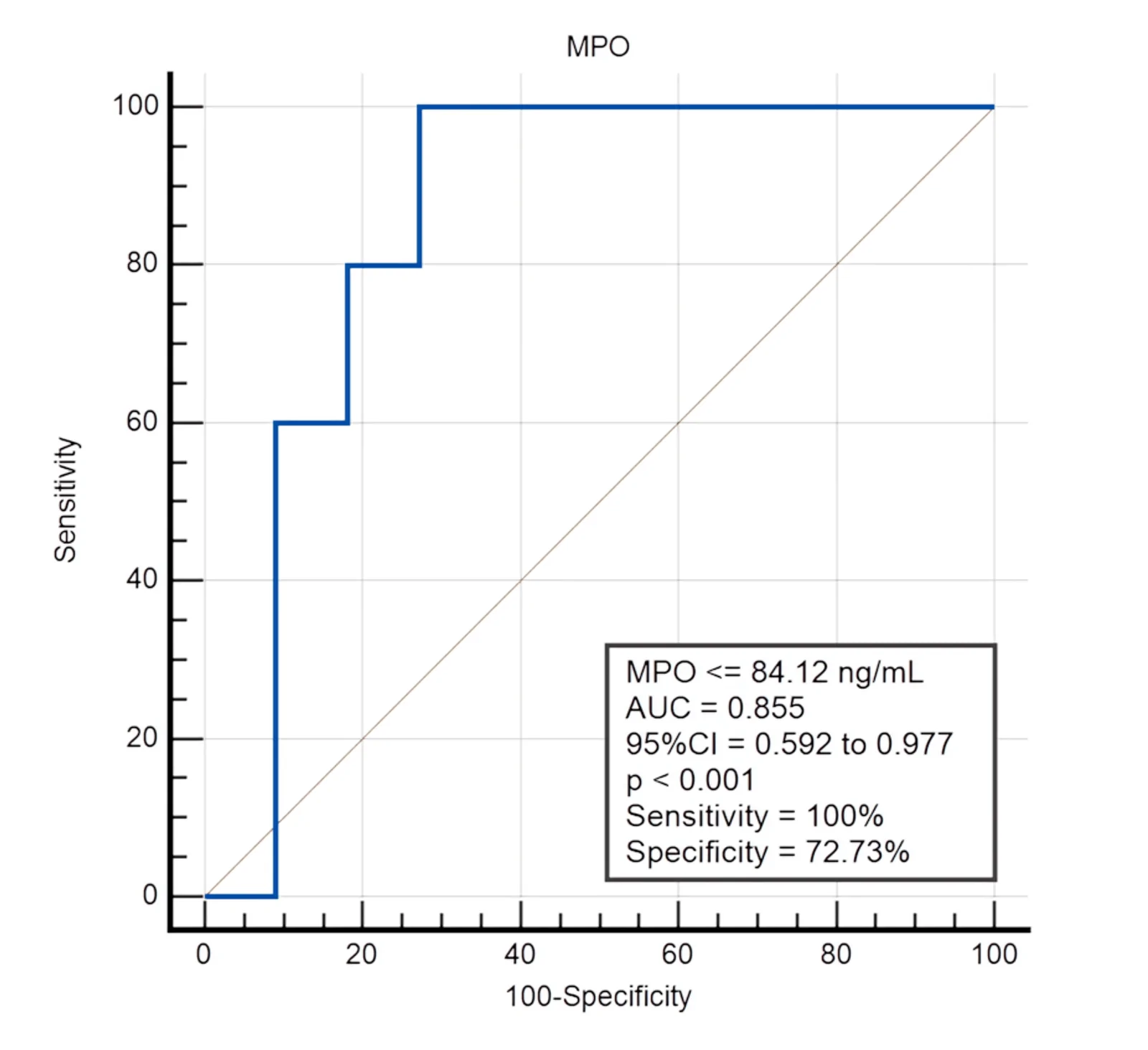
ROC curve analysis for discriminatory ability of MPO at T7 between animals with and without histopathologically detected thrombus AUC: area under the curve; 95% CI: 95% confidence interval; MPO: myeloperoxidase; ROC: receiver operating characteristic; T7: seven days after the intervention time point.

Among the evaluated biomarkers, MPO demonstrated the highest observed discriminatory performance for histopathologically detected thrombus (AUC = 0.855, 95%CI = 0.592 to 0.977, p < 0.001), with a cutoff of ≤ 84.12 ng/mL holding 100% sensitivity and 72.73% specificity (Figure 3).

**Figure 4:**
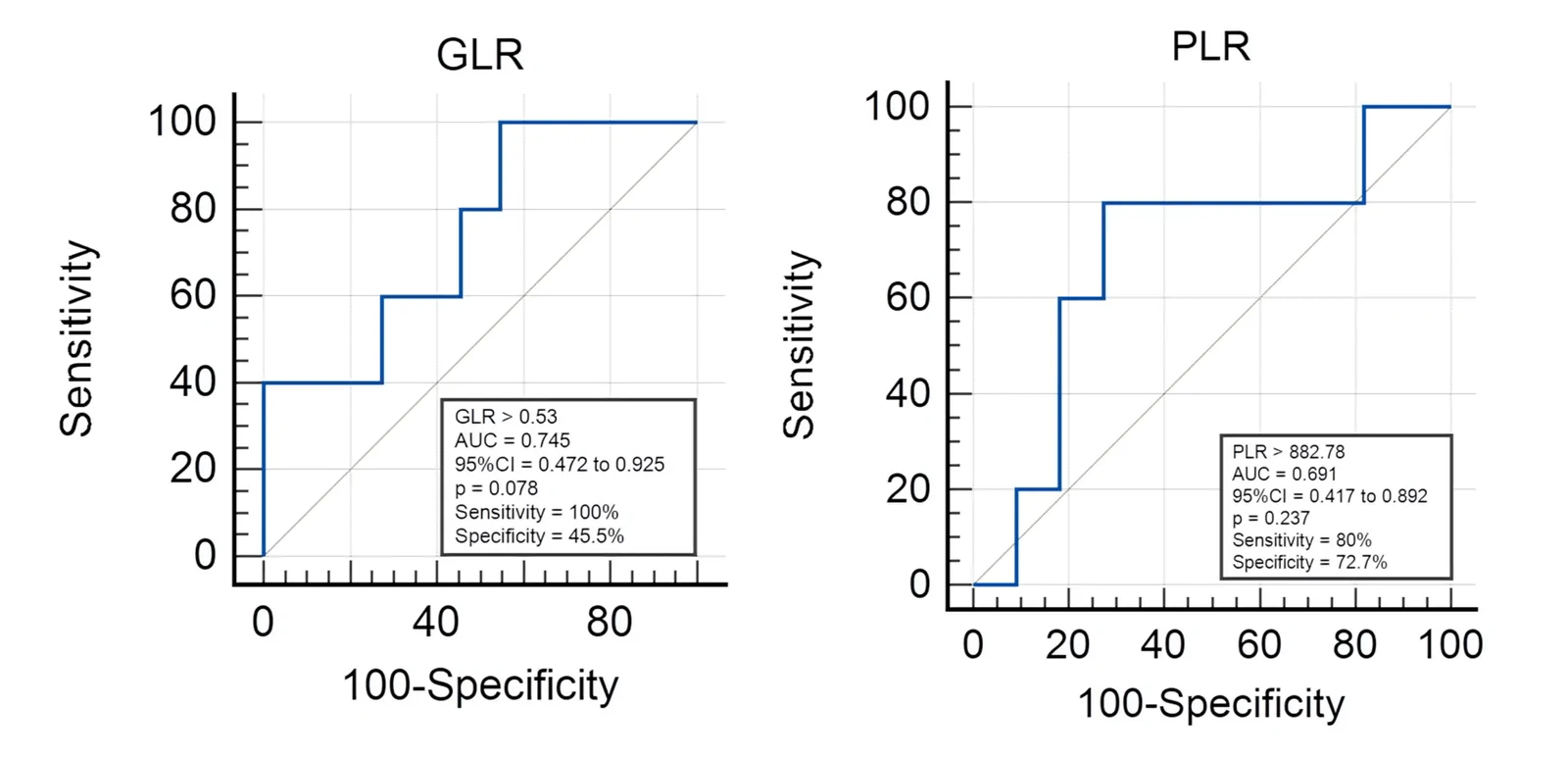
ROC curve analyses for discriminatory ability of GLR and PLR at T7 between animals with and without histopathologically confirmed thrombus AUC: area under the curve; 95% CI: 95% confidence interval; GLR: granulocyte-to-lymphocyte ratio; PLR: platelet-to-lymphocyte ratio; ROC: receiver operating characteristic; T7: seven days after the intervention time point.

ROC analysis suggested potential discriminatory ability for GLR (AUC = 0.745, 95%CI = 0.472-0.925) and PLR (AUC = 0.691, 95%CI = 0.417-0.892); however, neither marker reached statistical significance (p > 0.05) (Figure 4).

## Discussion

Although hematological parameters are occasionally reported in experimental models of venous thrombosis [23,24], they are generally not the primary focus of investigation. Consequently, the temporal dynamics of CBC parameters and derived inflammatory indices during thrombus formation and resolution remain poorly characterized. Our study explored the dynamic evolution of hematological parameters in a rat model of electrolytic femoral vein injury.

Normal variations in the CBC characteristics of rodents may occur depending on different variables such as age [25-27], sex [25-27], strain [25] (e.g., Sprague-Dawley rats [28] versus Wistar rats [26]), environmental conditions, including animal provenance [29], housing [26] and handling methods [25], diet [25]; or procedure-related factors such as anesthesia [25] and sampling site [25]. In our case, given the small sample size and to avoid potential confounders, we opted for the same strain, age, and sex (female) animals that were housed in shared cages and handled by the same personnel. Given the short duration of our experiment, variation between age at the beginning and at the end of the experiment should not have significantly impacted CBC parameters. However, the sampling site differed at the beginning of the experiment (caudal vein) versus the end of the experiment (intracardiac puncture), which may at least partially account for the significant differences found between baseline and study-end. Furthermore, the limited volume of blood that could be repeatedly collected over a short period of time without adversely affecting the physiological status of the experimental animals [30] restricted the number of sampling time points, thereby preventing a more detailed evaluation of temporal changes in CBC parameters.

Evaluation of CBC in our study revealed a statistically significant decrease in the absolute counts of leukocytes, granulocytes, and lymphocytes seven days after intervention in both sham and electrolytic injury treatment groups, proposing that the observed alterations in CBC could be due to postoperative inflammatory responses rather than electrolytic injury per se. Possible explanations for these observations could be the effects of endogenous corticosteroids released in response to surgical stress, which can cause lymphopenia [25], or neutrophil recruitment in the vascular wall [6], decreasing the number of circulating cells.

Indices derived from CBC analysis reflect the dynamics of circulating immune cell populations. While indices such as the neutrophil-to-lymphocyte ratio have been widely investigated in human deep vein thrombosis studies [7], other markers, including the granulocyte-to-lymphocyte ratio, remain largely unexplored, especially in experimental settings. Addressing these gaps may improve the understanding of thromboinflammatory responses and the translational relevance of animal models.

The GLR decreased significantly in the sham group, while it remained largely unchanged in both anticoagulation arms at T7 compared to T0. This GLR dynamic could be accounted for by a rather proportional late decrease of both granulocyte and lymphocyte counts in cases receiving treatment after the electrolytic injury.

Given that neutrophils constitute the majority of circulating granulocytes, in human studies GLR may approximate NLR; however, murine models present particular representations of cell lines. Compared to other rodent species that have predominant neutrophil populations, the leukocyte population of laboratory rats is characterized by lymphocyte predominance (and even surpassing granulocyte counts) [25], confirmed by our findings, where GLR was less than or equal to one, regardless of the experimental time point when it was determined. These differences make the establishment and interpretation of CBC-derived indices across species particularly challenging, limiting the translation of animal experimental findings regarding CBC-derived indices to human studies. Therefore, raising awareness regarding interspecies differences in leukocyte distribution and immune responses is important when considering animal models for research questions involving the hematological response to a proposed model of disease.

Platelet count increased in the enoxaparin group at T7 compared to T0, while in the rivaroxaban group no significant variation could be identified. PLR increased significantly in both groups, showing a progressive rise in the enoxaparin group and a delayed increase at T7 compared to T1 in the rivaroxaban group. However, comparison of rivaroxaban versus enoxaparin treated arms did not show any statistically significant difference in GLR and PLR between the two anticoagulation regimens.

Plasma MPO concentrations showed temporal changes in both treatment groups. In the rivaroxaban group, MPO increased significantly compared with baseline at both T1 and T7, whereas in the enoxaparin group an overall time effect was detected without significant pairwise differences between time points. Despite these within-group observations, no statistically significant differences in MPO concentrations were found between treatment groups at any time point. Taken together, these findings suggest that, within the limitations of this exploratory study, the overall MPO response was broadly comparable between the two anticoagulant regimens.

Published literature has described possible interactions between circulating MPO levels and anticoagulant treatments. A 2020 study on thrombosis prevention in cancer patients suggested that MPO release may partially reduce the anticoagulant efficacy of low molecular weight heparins like dalteparin and tinzaparin, but that it does not affect rivaroxaban [17]. Another report has determined that heparin mobilizes MPO from vascular compartments, therefore leading to increased plasmatic levels, but the related study was conducted on coronary arteries in patients undergoing coronary angiography [31].

The recent International Vascular Surgery Survey data supports a growing interest in rivaroxaban use within vascular and thromboinflammatory settings [32]. Preclinical and clinical studies suggest that rivaroxaban not only reduces coagulation biomarkers, but may also modulate platelet and endothelial activation, respectively, and inflammatory biomarkers [33]. A 2023 study on mice in homeostatic conditions and in response to cardiac ischemia showed a reduction of mature leukocytes in the bone marrow after one week of rivaroxaban treatment, as well as lowered circulating leukocytes after induced cardiac ischemia/reperfusion [34]. A 2025 study on rivaroxaban combined with low-dose aspirin in patients with cardiovascular disease showed a significant decrease in platelet-derived and MPO-positive extracellular vesicles at six months combined treatment follow-up when compared to baseline (aspirin only) [35]. However, the measurement of plasma free MPO does not capture MPO contained within extracellular vesicles, which can represent a biologically relevant pool of MPO released from activated neutrophils or other cells.

The 16 animals from the treated groups euthanised at T7 were included in the previous biomarker analyses. As an additional exploratory analysis, these animals were subsequently stratified according to thrombus status at T7 to explore whether the presence or absence of histopathologically detected thrombus was associated with differences in GLR, PLR, and MPO levels. Although GLR, PLR, and MPO levels did not differ significantly between animals with and without histopathologically detected thrombus, exploratory ROC analysis suggested potential discriminatory ability of MPO. ROC curve analysis determined an MPO threshold of <84.12 ng/mL suggestive of thrombus detection after six days of anticoagulation (AUC = 0.855, 95% CI = 0.592-0.977, p < 0.001, 100% sensitivity, ~73% specificity). These exploratory subgroup analyses should be interpreted cautiously, given the limited number of specimens with detected thrombus (5/16). Nevertheless, they do provide additional context regarding the relationship between inflammatory biomarkers and thrombus persistence.

Although MPO showed good discriminatory ability for histopathologically detected thrombus at study end, the lack of advanced imaging techniques to monitor thrombus formation, evolution, and resorption prevented the distinction between unsuccessful thrombus induction and complete thrombus resolution during anticoagulant treatment. The inconsistent thrombus formation observed in the present study may reflect the challenges associated with adapting an electrolytic venous injury protocol from mice to rats. Previous research on mice undergoing a similar protocol (positive current at 3 V applied for 90 s) showed thrombus persistence at seven days, with evolution towards stenotic fibrotic remodelling at 14 and 28 days [21]. However, in our rat model, hematoxylin-eosin staining of the venous samples identified thrombus formation in one of four untreated control animals at T1, indicating that the adapted protocol was capable of inducing thrombosis in at least some animals, while also highlighting variability in thrombus formation under the selected experimental conditions. In the electrolytic injury treated group, thrombus was detected at T7 in only five of 16 specimens. Because histopathological assessment was performed only at the study endpoint, it was not possible to determine whether the absence of thrombus reflected unsuccessful induction, thrombus resolution during anticoagulant treatment, or a combination of both. Accordingly, histopathological evidence of thrombus was obtained in at least 6 of 20 animals.

Techniques for thrombus visualization in vivo, such as intravital microscopy [36], which would have allowed for better assessment of procedure success, were unfortunately unavailable at the time of the experiment. Future studies incorporating serial in vivo imaging would permit longitudinal evaluation of thrombus formation and resolution and help optimize the reproducibility of the adapted protocol. Larger studies with further optimization and validation of the electrolytic injury model in Wistar rats are recommended.

Study limitations and future directions

While the study has important limitations that we will discuss in the following paragraphs, we consider that the current findings, although exploratory, are relevant for future studies that may further adapt and validate the electrolytic model of femoral vein injury as a reproducible thrombosis model.

Our adapted protocol showed limited evidence of venous thrombosis under the presented experimental conditions, based on the histopathological evaluation performed at the prespecified endpoints. Thrombotic material was identified by histopathological analysis in 1/4 injury control samples and 5/16 injury control treated animals. Consequently, interpretation of thromboinflammatory biomarkers in our experimental animals should be made cautiously, as some observed changes may reflect responses to vascular injury rather than modifications related to thrombotic processes. However, given the unavailability of advanced imaging techniques to confirm thrombus formation and evolution at the time we performed this study, thrombus induction success cannot be appropriately assessed. Furthermore, treatment administration may have contributed to the resorption of small thrombi, while microembolization before the prespecified endpoints cannot be excluded. Therefore, future therapeutic studies using this model should preferably incorporate at least one in vivo imaging modality to confirm thrombus formation and monitor its evolution before and during therapeutic intervention.

Other relevant limitations of this study include the small sample size of the experimental groups, which reduces statistical power, particularly for detecting small between-group differences, and the lack of additional control groups with the same prespecified endpoints. Expanding the sample size and including additional control groups, such as non-operated or treatment administration vehicle-control groups, might provide a better overview of potential methodological sources of bias. While application of the 3Rs (Replacement, Reduction, and Refinement) is of foremost importance in animal research, overly small sample sizes may increase the influence of inter-animal variability and limit the representativeness of the study population.

Potential sources of bias should also be considered, including differences in baseline biological parameters, sequential allocation, effects of anesthesia and surgical manipulation, operator-related variability and learning-curve effects, lack of blinding during procedural steps, different sampling sites throughout the experiment, and differences in treatment exposure related to the routes of administration.

In our research, sequential allocation may have led to temporal and operator-related bias, including a potential learning-curve effect, especially given the first implementation of this adapted animal model in our facility. The partial blinding, which was limited to histopathological analysis, and the lack of blinding during procedural steps, including group allocation, treatment administration, and blood sampling, may have introduced operator-related bias.

Furthermore, variation in sampling sites and differences in prespecified endpoints limited both intra-group and inter-group comparisons. Differences in sampling procedures may be particularly relevant for CBC-derived inflammatory indices, and therefore, inter-group comparisons were restricted to groups with comparable sampling procedures, where appropriate. In particular, the electrolytic injury control group was not included in some comparisons because of differences in sampling procedures; for MPO, this group was additionally not assessed because of limited assay resources. Additionally, our study focused on a relatively limited set of circulating biomarkers, reflecting the need to balance the number of assays with the recommended limits for repeated blood sampling in experimental animals. The required sample volume for individual assays, particularly ELISA-based measurements, should therefore be considered during study design, together with the expected plasma or serum resulting from the planned sampling volume. These factors should be accounted for when selecting biomarkers to be further evaluated, preferably through standardized sampling procedures across groups.

Differences in systemic drug exposure related to the administration route, more specifically in the case of subcutaneous versus oral administration, represent another potential source of bias, alongside the pharmacological properties of each medication. Rivaroxaban was administered orally using a palatable food vehicle to facilitate voluntary intake and minimize stress, as well as to reduce possible bleeding related to gavage-administered oral anticoagulation. While this approach improved compliance, variability in oral intake and drug absorption cannot be excluded. The lack of plasma anti-Xa activity measurements and plasma drug concentration data does not allow pharmacokinetic confirmation of equivalent drug exposure or therapeutic anticoagulation. Additionally, the use of Nutella as the administration vehicle may have influenced inflammatory biomarkers through transient metabolic effects, including postprandial hyperglycemia. Consequently, a potential confounding effect of the oral vehicle on inflammatory marker levels cannot be ruled out. These methodological constraints should be taken into account when interpreting between-group differences. Future research directions should also include pharmacokinetic and pharmacodynamic assessments, including plasma rivaroxaban concentrations and anti-Xa activity, to confirm drug exposure and allow a more direct evaluation of the relationship between anticoagulation and thromboinflammatory responses.

For histopathological analysis, although blinding was applied to reduce operator-related bias, immunohistochemical markers (e.g., fibrin, platelet, or NET-associated markers) were not included in our research due to the exploratory nature of the study and the limited availability of tissue, which was fully processed for routine histological evaluation. Future studies should aim to expand the histopathological assessment while considering methodological constraints, for a better characterization of the thromboinflammatory burden in similar study designs.

The results of our analyses should therefore be considered exploratory and hypothesis-generating rather than confirmatory. The current findings should be interpreted within the specific experimental conditions of this study. Accordingly, the proposed MPO threshold should be regarded as preliminary and hypothesis-generating rather than as a validated diagnostic cutoff. In particular, the limited number of animals with histologically confirmed thrombotic material and the absence of a definitive method for confirming thrombus formation and evolution in vivo limit the interpretation and external validity of the proposed MPO threshold.

These limitations should be addressed in future studies to strengthen the validity and reproducibility of the findings. Further optimization and validation of the protocol in rats are required before its broader application as a thrombosis model.

To conclude, the main recommendations for future experimental designs include larger and more balanced groups; randomized and concurrent allocation where feasible; standardized and concurrent sampling procedures; appropriate sham, untreated, and vehicle controls; blinded assessment where feasible; and direct confirmation of thrombus formation using in vivo imaging and/or validated histopathological methods. Broader biomarker panels should be implemented together with tissue-based analyses to better distinguish vascular injury, inflammation, and thrombosis. An example of an expanded biomarker panel includes quantitative assessment of C-reactive protein, pro-inflammatory cytokines (e.g., interleukins IL-1 and IL-6), and markers of thromboinflammation and immunothrombosis, such as extracellular DNA-MPO and DNA-neutrophil elastase complexes as indicators of NETosis. These approaches would improve reproducibility, reduce potential sources of bias, and strengthen the generalization potential of the findings.

## Conclusions

This exploratory study characterized inflammatory and hematological responses following an adapted electrolytic femoral vein injury procedure in rats. Significant temporal changes in CBC parameters were observed, including a significant late (seven days after the procedure) decrease in leukocyte, granulocyte, and lymphocyte counts, together with a significant increase in the PLR. Among the evaluated biomarkers, MPO showed the highest observed discriminatory performance for histopathological thrombus detection, suggesting its potential as a thromboinflammatory biomarker.

Future research should consider refining the experimental model, broadening the biomarker panel, and incorporating advanced in vivo and ex vivo approaches for thrombus evaluation to further elucidate the pathophysiological processes underlying thromboinflammation.
